# Standardization and quality assessment for human intestinal organoids

**DOI:** 10.3389/fcell.2024.1383893

**Published:** 2024-09-12

**Authors:** Hana Lee, Seunghye Yang, Kyung Jin Lee, Si-Na Kim, Ji-Seon Jeong, Ki Young Kim, Cho-Rok Jung, Sooyeon Jeon, Dayeon Kwon, Sungin Lee, Hanbyeol Lee, Chihye Park, Sun-Ju Ahn, Jongman Yoo, Mi-Young Son

**Affiliations:** ^1^ Korea Research Institute of Bioscience and Biotechnology (KRIBB), Daejeon, Republic of Korea; ^2^ Organoid Standards Initiative (OSI), Department of Biophysics, Institute of Quantum Biophysics, Sungkyunkwan University, Suwon, Republic of Korea; ^3^ ORGANOIDSCIENCES, Seongnam-si, Republic of Korea; ^4^ Korea Research Institute of Standards and Science (KRISS), Daejeon, Republic of Korea; ^5^ Therapeutics and Biotechnology Division, Korea Research Institute of Chemical Technology, Daejeon, Republic of Korea; ^6^ KRIBB School of Bioscience, Korea University of Science and Technology (UST), Daejeon, Republic of Korea; ^7^ Digital Health Laboratory, Department of Biophysics, Sungkyunkwan University, Suwon, Republic of Korea; ^8^ Department of Microbiology, CHA University School of Medicine, Seongnam-si, Republic of Korea; ^9^ Department of Biological Science, Sungkyunkwan University, Suwon, Republic of Korea

**Keywords:** standardization, guideline, human adult stem cell (hASC), human pluripotent stem cell (hPSC), human intestinal organoid (hIO)

## Abstract

To enhance the practical application of intestinal organoids, it is imperative to establish standardized guidelines. This proposed standardization outlines a comprehensive framework to ensure consistency and reliability in the development, characterization, and application of intestinal organoids. The recommended guidelines encompass crucial parameters, including culture conditions, critical quality attributes, quality control measures, and functional assessments, aimed at fostering a standardized approach across diverse research initiatives. The implementation of these guidelines is anticipated to significantly contribute to the reproducibility and comparability of results in the burgeoning field of intestinal organoid research.

## 1 Introduction

These guidelines aim to offer insights into the practical applications of intestinal organoids. The methods and efficacy of organoid production vary based on the target organ or tissue, with variations stemming from the cellular origin—human adult stem cells (hASCs) or human pluripotent stem cells (hPSCs). We aimed to establish terminology, definitions, production methods, and characteristic assessment approaches for the quality management of human intestinal organoids derived from hASCs or hPSCs.

### 1.1 Rationale

The increasing demand for alternative testing methods has expanded the application scope of novel cell models, resulting in the proliferation of diverse assays and service development using organoids (Almeqdadi et al., 2019; Zhao et al., 2022; Singh et al., 2023). Specifically, the active development of patient-specific and personalized human organ platforms using organoids is significant. These platforms extend beyond the fields of drug discovery, toxicology, cosmetics, and health-functional food screening to the field of regenerative medicine (Barker, 2014; Clevers, 2016; Kim et al., 2020; Okamoto et al., 2020; Kasendra et al., 2021; Sugimoto et al., 2021; Wang Q. et al., 2022; Urbano et al., 2022; Takahashi et al., 2023; Tian et al., 2023). Organoids serve as tissue-engineered, cell-based, *in vitro* models that mimic the structure and function of tissues within the body (Meran et al., 2020; Nikolaev et al., 2020; Rezakhani et al., 2021; Gjorevski et al., 2022; Meran et al., 2023). These models have been extensively studied as alternatives to animal experimentation, offering safe and accurate predictions of the effects of drugs on the human body.

However, the lack of standards for organoid production and quality management poses significant limitations in the transition to clinical and other applied fields. Therefore, recognizing the specificity of intestinal organoids derived from hASC or hPSC, these guidelines have been published to present considerations essential for manufacturing a standardized human intestinal organoid model and assessing its quality.

### 1.2 Principle and scope of application

#### 1.2.1 General principles

Organoids derived from hASC or hPSC comprise various cell types that exist in complex mixtures with diverse differentiation capabilities and cells at various stages of differentiation (Sato et al., 2009; Spence et al., 2011; Sato and Clevers, 2013; Barker, 2014; Fujii et al., 2018; Duckworth, 2021). The *in vivo* differentiation potential and mechanisms of action of organoids can vary based on culture conditions and the duration of *in vitro* culture. Factors, such as medium composition (use of growth factors or serum), isolation methods, cell size, and confluence can affect cell composition and biological activity.

**FIGURE 1:**
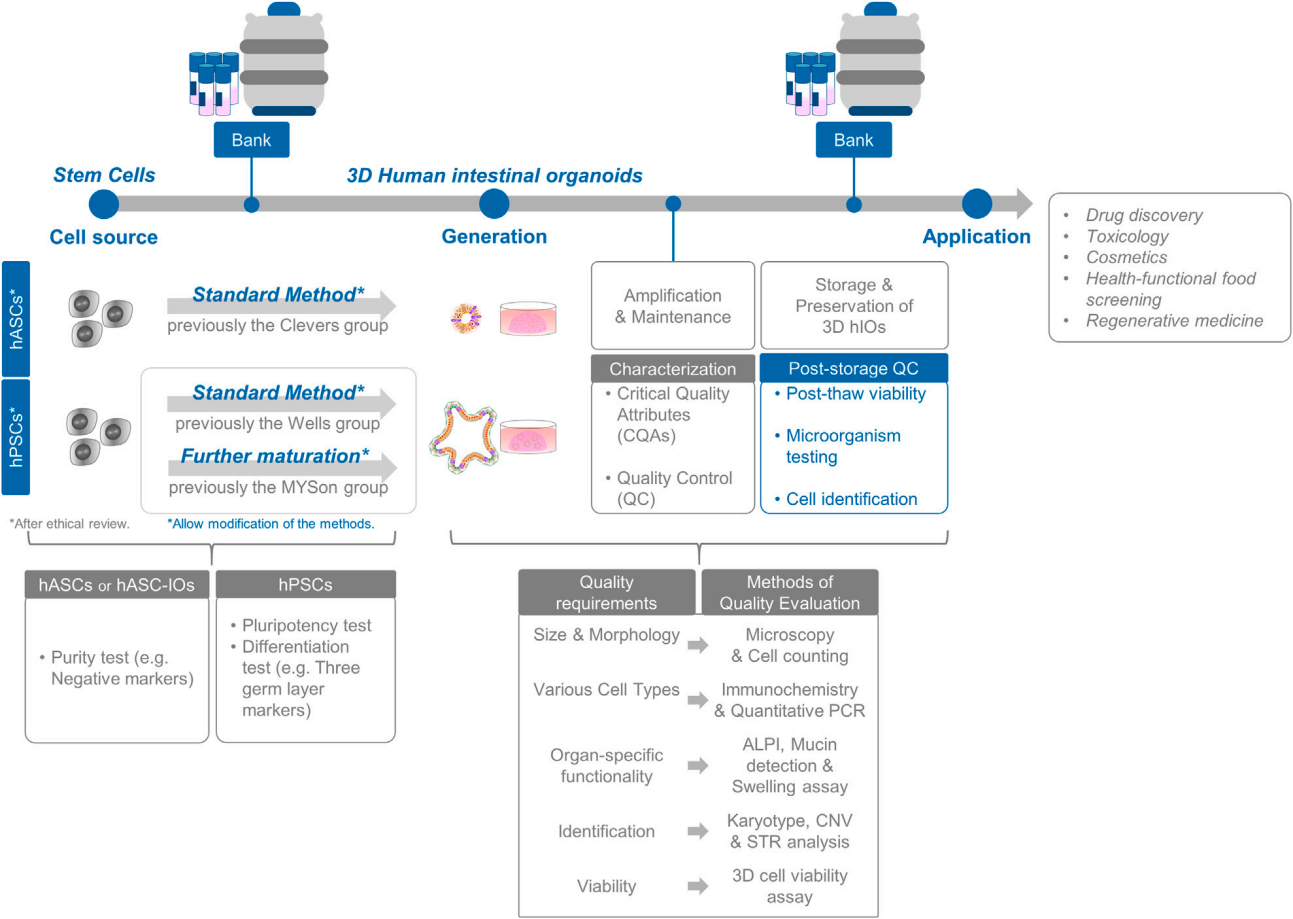
Standardization and assessment of intestinal organoid production. The process encompasses the selection of cell sources and comprehensive *in vitro* characterization of cell preparations. Evaluation of intestinal organoids involves a thorough analysis of organoid functionality and identity, along with rigorous post-storage quality control measures for banking purposes.

The differentiation flexibility and product-specific characteristics of stem cell-based organoids are imperative for conducting alternative testing methods, nonclinical studies, and clinical research using well-defined and characterized organoids. Additionally, the support matrix (Matrigel) used for three-dimensional (3D) culture plays a structural role in facilitating the growth of new tissues (Sato et al., 2009; Barker et al., 2010; Hughes et al., 2010; Spence et al., 2011). While exploring clinical applications, novel perspectives may emerge for the assessment of regenerative therapies, 3D organoids, and support matrices. Although organoids used in basic research, assessment models, and non-clinical processes require separate assessment of their product components, a systematic assessment of the characteristics and actions of hASC- or hPSC-derived organoids with support matrices is essential (see Figure 1).

#### 1.2.2 Scope of application

The term “intestinal organoid” refers to a product that is either directly differentiated from hPSCs (embryonic stem cells or induced pluripotent stem cells) or that uses live hASCs. These products incorporate scaffolds, cell cultures, and proliferation processes to develop 3D organoids that can mimic organs.

Current production methods for enteric organoids have been developed individually based on whether they are derived from hASCs or hPSCs. This guidance aims to offer specific details on common production methods based on cell sources, which are crucial for the standardized production of products for practical applications. These guidelines can be applied to cell-supported matrix composite products that are categorized as assessment models for the development of cell therapies or alternative testing methods.

### 1.3 Term definitions

#### 1.3.1 Human adult stem cell (hASC)

Adult stem cells are undifferentiated cells present in specific differentiated tissues of the body. They exhibit the potential to generate new cells capable of self-renewal or replenishing damaged or deceased tissues (Clevers and Watt, 2018; Post and Clevers, 2019; Beumer and Clevers, 2024).

#### 1.3.2 Human pluripotent stem cell (hPSC)

Pluripotent stem cells (PSCs) are usually present during early embryonic developmental stages. They exhibit the potential to differentiate into three germ layers (ectoderm, endoderm, and mesoderm) and into germ cells. The hPSCs comprise both human embryonic stem cells (hESCs) and human induced pluripotent stem cells (hiPSCs) (Keller, 1995; Desbaillets et al., 2000).

#### 1.3.3 Human embryonic stem cell (hESC)

Embryonic stem cells, located in the inner cell mass of human blastocysts during the early embryonic development exhibit pluripotency and form three germ layers (Thomson et al., 1998).

#### 1.3.4 Human induced pluripotent stem cell (hiPSC)

Induced pluripotent stem cells (iPSCs) are derived from somatic cells through genetic reprogramming using the forced expression of genes or dedifferentiation factors (Yamanaka factors: octamer-binding transcription factor 4 [Oct3/4], sex determining region Y-box 2 [Sox2], Kruppel-like factor 4 [Klf4], and cellular-myelocytomatosis [c-Myc]), which resemble embryonic stem cells (Takahashi et al., 2007).

#### 1.3.5 Pluripotency

The potential of stem cells to differentiate into cells belonging to the three germ layers (ectoderm, endoderm, and mesoderm) and germ cells (Yilmaz and Benvenisty, 2019).

#### 1.3.6 Differentiation

Transformation of stem cells into specific cell types with distinct functions (Sanchez Alvarado and Yamanaka, 2014).

#### 1.3.7 Definitive endoderm (DE)

It is formed when precursor cells from the early endoderm, located in the epiblast, invade and form a layer beneath it. These DE precursor cells generate all endodermal tissues, including the intestine (de Santa Barbara et al., 2003; D'Amour et al., 2005).

#### 1.3.8 Hindgut (HG)

The HG includes the posterior (tail) portion of the digestive tract in mammals, including the upper third of the transverse colon, descending colon, sigmoid colon, and rectosigmoid junction (Wells and Melton, 2000; Dessimoz et al., 2006; Spence et al., 2011).

#### 1.3.9 Organoid

Self-organizing 3D structures derived from stem cells (pluripotent or adult) in a laboratory setting can mimic the cellular diversity, structure, and specific functions of tissues within the body (Lancaster and Knoblich, 2014; Takebe and Wells, 2019).

#### 1.3.10 Human intestinal organoid (hIO)

An intestinal organoid derived from hASC or hPSC, capable of self-organization and partially mimicking the identity, cellular diversity, and functionality of the human intestine (Sato et al., 2009; Sato et al., 2011a; Spence et al., 2011; Sato and Clevers, 2013).

#### 1.3.11 Mature human intestinal organoid (Mat-hIO)

It is specifically derived from the direct differentiation of hPSC, which distinguishes them from the general characteristics of immature fetal tissues inherent in hPSC-derived intestinal organoids. To foster enhanced maturation and attain advanced cellular diversity and functionality in these intestinal organoids, distinct niche factors (interleukin-2 [IL-2], IL-22, NRG1, and IGF-1/FGF-2) have been introduced into the culture environment (Fujii et al., 2018; Jung et al., 2018; Jarde et al., 2020; Holloway et al., 2021; He et al., 2022).

#### 1.3.12 Passage

The process of dividing existing organoids into smaller fragments or individual cells and maintaining their growth in a laboratory environment (Ganesh et al., 2019; Pleguezuelos-Manzano et al., 2020).

#### 1.3.13 Cryopreservation

The process of preserving organoids in a dormant state at low temperatures, ensuring the retention their cell composition, gene expression, and functional characteristics (Pleguezuelos-Manzano et al., 2020; Lee et al., 2022).

#### 1.3.14 Thawing

Transition of frozen organoids from dormant state to an active growth state.

#### 1.3.15 Intestinal stem cell (ISC)

ISCs are located in the basal region of small intestinal crypts. They exhibit both self-renewal and differentiation abilities, thereby giving rise to various intestinal epithelial cell types (Barker et al., 2012; Clevers, 2013; Barker, 2014).

#### 1.3.16 Intestinal stem cell (ISC) differentiation

The process in which ISCs divide into daughter cells and differentiate into various cell types, including enterocytes, goblet, Paneth, enteroendocrine, and transit-amplifying cells (de Santa Barbara et al., 2003; Gehart and Clevers, 2019; Beumer and Clevers, 2021; Duckworth, 2021; Holloway et al., 2021).

#### 1.3.17 Transit-amplifying cell (TA cell)

TA cells are derived from stem cells that possess significant proliferative capacity and later differentiate into various types of mature intestinal epithelial cells (Hsu et al., 2014; Gehart and Clevers, 2019).

#### 1.3.18 Enterocyte

These intestinal epithelial cells are primarily responsible for nutrient absorption in the intestinal lumen (Doherty and Charman, 2002; International Transporter et al., 2010).

#### 1.3.19 Goblet cell

These cells periodically secrete mucus in the gastrointestinal tract and are characterized by goblet-shaped mucus-filled granules at the top and nuclei at the bottom (Chang et al., 1994; Gustafsson and Johansson, 2022).

#### 1.3.20 Paneth cell

These cells are located at the base of the small intestinal crypts, comprising thick eosinophilic granules at the top and a conical shape with a rounded nucleus at the bottom (Porter et al., 2002; Bevins and Salzman, 2011; Sato et al., 2011b).

#### 1.3.21 Enteroendocrine cell

These cells in the intestinal tract secrete intestinal hormones in response to food stimulation or pH changes and are characterized by irregularly shaped cone-like cells with numerous secretory particles at the base (Gribble and Reimann, 2019; Zeve et al., 2022).

## 2 General considerations

### 2.1 Cell source

#### 2.1.1 General recommendations

Starting materials including hASCs and hPSCs, are identified based on the International Society for Stem Cell Research (ISSCR) Standards for Human Stem Cells Use in Research (https://www.isscr.org/standards). Additionally, these guidelines aim to propose specific and detailed quality control indicators to enhance the reproducibility of the human intestinal organoid model system, ensuring alignment with the rigorous standards set forth by the ISSCR.

##### 2.1.1.1 Adult stem cell-derived organoids

The hASC-derived organoids were produced from live intestinal tissues obtained from the donors. Therefore, it is crucial to establish the origin and source of these tissues. To demonstrate the suitability of donor tissues for organoid production, donor eligibility should be assessed through medical history assessments, blood tests, and microbiological examinations. When hASC-derived intestinal organoids are collected from donor tissues, they comprise specific cell groups (ISCs) during the passage cultivation process, thereby organizing into organoids. Therefore, during isolation and culture, it is crucial to confirm the characteristics of target cells specific to the intestinal crypts. These characteristics should be maintained until the final step or the establishment of the cell bank. Additionally, to ensure the maintenance of purity, test items capable of confirming impurities in non-target cells should be established. If donor cells or tissues need to be transported after collection for production, appropriate transport conditions (preservation solution, temperature, and time) should be set, and stability during transportation should be assessed.

##### 2.1.1.2 Pluripotent stem cells

The origin and source of the cells before differentiation are crucial for the use of hPSCs. The characteristics of hPSCs should be confirmed, because organoids are produced by mimicking the differentiation process of hPSCs (embryonic and iPSCs) to resemble human development. Therefore, to verify the suitability of the source cells for differentiation, test items capable of confirming pluripotency should be established, and karyotype analysis should be performed to ensure chromosomal normalcy.

#### 2.1.2 Quality management recommendations

The following test items are recommended based on the purpose of using the intestinal organoids.

##### 2.1.2.1 Adult stem cell-derived organoids

For adult stem cell-derived organoids, donor suitability blood tests are advised for the detection of Human Immunodeficiency Virus (HIV), Hepatitis B Virus (HBV), Hepatitis C Virus (HCV), and syphilis. Additionally, microbiological tests, such as sterility testing, *Mycoplasma* negative test, and endotoxin testing were advised.

###### 2.1.2.1.1 Purity tests

Various tissues and cells, such as blood vessels, muscles, nerve fibers, and white blood cells, are present near the intestinal crypts. Therefore, it was impossible to eliminate the possibility of introducing unwanted cells during the tissue collection. The cells that may be introduced during the collection of intestinal tissues, include fibroblasts, mesenchymal cells, vascular cells, and blood cells. These cells are designated as “unwanted cells,” and their purity is verified by selecting appropriate antibody markers (negative markers, see Table 1) and using methods, such as flow cytometry.

**TABLE 1:** Negative markers for purity test in ASC-derived organoids.

Negative markers	
FOXD3	Enteric glia cells
alpha SMA, Cofillin 2	Smooth muscle cells
Vimentin, Decorin, Fibulin 1	Fibroblasts
C1QA, CD14, CD68, CD86, CYBB	Macrophages
FCAR, ADAMTS4, AQP9, GPR3	Neutrophils
CPA3, ERVFRD-1, GCSAML, RHEX, SIGLEC6, SLC18A2	Mast cells
IGHA1, IGKC, IGLC1, JCHAIN	Plasma cells
HLA-DR, CD40, CD207, CD304, CD49d, CD1c, CD197, CD86, CD1a, CD1b, CD80, CD11b, CD205, F4/80, CD273, CD11c, CD209, CD83, MHC class II	Dendritic cells
CD45, CD3, CCR6, FOXP3	T cells
CD31	Endothelial cells

##### 2.1.2.2 Pluripotent stem cells

For pluripotent stem cell-derived organoids, microbiological tests, such as sterility testing, *Mycoplasma* negative test, and endotoxin testing were performed.

###### 2.1.2.2.1 Pluripotency tests

To confirm pluripotency, specific stem cell markers were assessed using immunostaining or qPCR (Andrews and Gokhale, 2024) (see Table 2).

**TABLE 2:** Stem cell markers.

Stem cell markers	
OCT4	NANOG
TRA-1-60	TRA-1-81
SSEA-3	SSEA-4

###### 2.1.2.2.2 Differentiation tests of three germ layers (ectoderm/endoderm/mesoderm)

To confirm differentiation, markers specific to the ectoderm, endoderm, and mesoderm were assessed using immunostaining or qPCR (see Table 3). Additionally, differentiation into the three germ layers was assessed using teratoma formation *in vivo*, which served as an assessment method for confirming the differentiation process (Son et al., 2017).

**TABLE 3:** Three germ layer markers.

Three germ layer markers	
TUJ1, NESTIN	Ectoderm
FOXA2, SOX17	Endoderm
DESMIN, α-smooth muscle actin (α-SMA)	Mesoderm

#### 2.1.3 Cell types and characteristics

##### 2.1.3.1 Adult stem cell-derived organoids

Adult stem cell-derived intestinal organoids undergo continuous culture and differentiation after the isolation of crypts from the colon tissue. As the passages progressed, specific hASCs-derived cell groups were organized into organoids. Additionally, during the culture of intestinal organoids, there was no direct contact with the other cell types. Therefore, the characteristics of the source cells can be inferred from the characteristics of the intestinal organoids after culturing in Section 2.2 (Culture). The characteristics of the intestinal organoids are detailed in Section 2.3 (Quality Requirements and Assessment).

##### 2.1.3.2 Pluripotent stem cell-derived organoids

hPSCs demonstrated unlimited proliferation during passaging, without altering their properties. The characteristics of the source cells are described in Section 2.1 (Cell Source). hPSC-derived intestinal organoids undergo direct differentiation and culturing processes in Section 2.2 (Culture). The characteristics of these organoids are detailed in Section 2.3 (Quality Requirements and Assessment).

### 2.2 Culture

Note: The essential elements and culture procedures described below adhere to the primary, currently well-proven protocols for generating intestinal organoids (Sato et al., 2009; Spence et al., 2011; Jung et al., 2018; Pleguezuelos-Manzano et al., 2020). Organoids produced according to the recommended protocol must meet the standards outlined in Section 2.3. This underscores the utilization of established protocols as benchmarks for producing intestinal organoids that meet quality requirements, while allowing for minor modifications as needed.

#### 2.2.1 Adult stem cell-derived organoids

##### 2.2.1.1 Essential elements

###### 2.2.1.1.1 Media components

####### 2.2.1.1.1.1 Basal medium

Basal medium was prepared using advanced Dulbecco’s Modified Eagle Medium/F12 (advanced DMEM/F12) supplemented with 10 mM N-2-hydroxyethylpiperazine-N′-2-ethanesulfonic acid (HEPES) buffer, 1× GlutaMAX Supplement, 100 U/mL penicillin/streptomycin (and/or Primocin), and 10% (v/v) fetal bovine serum (FBS). It can be stored at 4°C for up to 1 month.

####### 2.2.1.1.1.2 Expansion medium

The basal medium is supplemented with B-27 supplement, 50% (v/v) Wnt3A-CM or 0.5 nM Wnt surrogate (U-Protein Express, N001), 20% (v/v) Rspo1-CM, 2% (v/v) noggin conditioned medium (U-Protein Express, N002), 50 ng/mL epidermal growth factor (EGF), 1.25 mM N-acetylcysteine, 10 mM nicotinamide, 10 μM p38 mitogen-activated protein kinases (MAPK) inhibitor (SB202190), 0.5 μM activin receptor-like kinase 5 (ALK5) inhibitor (A83-01), and 1 μM prostaglandin E2 (PGE2). It can be stored at 4°C for up to 1 month.

###### 2.2.1.1.2 Growth factors

Recombinant human R-Spondin1 (Rspo1), recombinant human noggin, recombinant human EGF, N-acetylcysteine, nicotinamide, p38 MAPK inhibitor (SB202190), ALK5 inhibitor (A83-01), and prostaglandin E2 (PGE2).

###### 2.2.1.1.3 Reagents

Matrigel or other extracellular matrix (ECM) basement membrane extracts (BME) (Pleguezuelos-Manzano et al., 2020), advanced DMEM/F12, B-27 supplement, collagenase type II, Y-27632 Rho kinase inhibitor, HEPES, red blood cell lysis solution, and antibiotics (penicillin/streptomycin, gentamicin, and Primocin).

##### 2.2.1.2 Culture process and requirements

The culture process and requirements for hASC-derived organoids may vary based on the target organ, research objectives, and culture methods. This guideline offers representative examples of the practical applications of intestinal organoids.

###### 2.2.1.2.1 Culture protocols

Crypts were extracted from human biopsy tissues of the small intestine and colon. Subsequently, they were cultured in a dome shape using an expansion medium containing growth factors to facilitate organoid growth.

####### 2.2.1.2.1.1 Cell preparation

Our research complies with all relevant ethical regulations. All studies based on human adult stem cells were approved by the IRB at Bundang Cha Medical Center (IRB numbers: CHAMC 2020-02-014 and CHAMC 2020-04-019).

1. Tissue samples were stored in 50 mL tubes containing advanced DMEM/F12 and Primocin at 4°C until separation. To ensure optimal results, 30 mg of tissue was dissociated within 24 h to prevent cell death and freezing.
2. Fresh digestion medium was prepared for each tissue sample by adding 5 mL of advanced DMEM/F12 to a 15 mL tube and combining it with 5 mg/mL Collagenase Type II and 10 μM Y27632. The mixture was maintained at 4°C until further use.
3. Subsequently, the tissues were transferred to a 10 cm Petri dish.
4. The tissue was cut into ∼1 mm^3^ pieces using two scalpels, and 5 mL of digestion medium was added using a pipette.
5. The contents from the Petri dish were transferred to a 15 mL tube, and the lid was sealed using parafilm. The Petri dish was then rinsed with a digestion medium.
6. The tissues were shaken at 37°C and 140 rpm for 30–45 min for dissociation. Following dissociation (approximately 30 min later), a highly turbid solution with no significant clumps was observed.
7. The dissociated tissue was filtered into a 50 mL plastic tube using a 100 μm cell strainer.
8. The filtrate was centrifuged (450 × g, 4°C, 5 min), and the supernatant was separated. If red blood cells (RBCs) were present, a dark red pellet was observed. The pellet was resuspended in RBC lysis buffer (3 mL) and incubated at room temperature (RT) for 5 min. Following culturing, 5 mL of advanced DMEM/F12 was added to the cell suspension. The mixture was centrifuged (450 × g, 4 °C, 5 min) and the upper layer was separated.
9. The pellet was washed liquid nitrogen twice with 10 mL of advanced DMEM/F12 and further centrifuged (450 × g, 4°C, 5 min) to separate the supernatant.
10. The pellet was resuspended in the original solution and floated in ECM (BME).

####### 2.2.1.2.1.2 Intestinal organoid culture

1. The cell suspension was seeded on preheated cell culture plates.
2. The plates were inverted, incubated in a 5% CO_2_ cell culture incubator at 37°C, and allowed to stand for 20 min.
3. Culture medium (500 μL) supplemented with 10 μM Y27632 was added to each well, and the plate was cultured in an incubator.
4. Approximately 1 week later, mechanical splitting was performed for continuous culture and passage of organoids.

####### 2.2.1.2.1.3 Mechanical splitting, passage, and expansion of human intestinal organoids

1. The culture medium was withdrawn from the cultured organoids, washed with advanced DMEM/F12 (1 mL) maintained at 4°C, and the organoids were collected into a tube containing 4°C advanced DMEM/F12 using a pipette.
2. The collected organoids were centrifuged (450 × g, 4°C, 5 min), and the supernatant was separated.
3. Subsequently, advanced DMEM/F12 was added using a P1000 pipette, and the organoids were pipetted 5–10 times until no large clumps were visible in the pellet.
4. Centrifugation was repeated (450 × g, 4°C, 5 min), and the supernatant was separated.
5. The pellet was resuspended in 300–400 μL of BME (the final concentration of BME was 75%–100%).
6. The cell suspension was seeded into preheated cell culture plates.
7. The plates were inverted, incubated in a 5% CO_2_ cell culture incubator at 37°C, and allowed to stand for 20 min.
8. The expansion medium was added and the culture medium was changed every 2–3 days.
9. Approximately 1 week later, mechanical splitting was performed for organoid passage.

This process facilitates the self-organization of new organoids and the proliferation of stem cells and differentiated amplifying cells within the organoids, contributing to the formation of organoids. Mechanical splitting and passage were repeated to maintain the growth and characteristics of the organoids over time.

###### 2.2.1.2.2 Essential environmental conditions

The culture was maintained at 37°C in a 5% CO_2_ incubator at saturated humidity.

All culture steps were performed in a sterile environment to prevent contamination.

#### 2.2.2 Pluripotent stem cell-derived organoids

##### 2.2.2.1 Essential elements

###### 2.2.2.1.1 Media components

####### 2.2.2.1.1.1 Maintenance of hPSCs

The hPSCs were maintained in mTesR1 medium.

####### 2.2.2.1.1.2 Differentiation of hPSCs into DE

hPSCs were differentiated into DE by supplementing the Roswell Park Memorial Institute (RPMI) 1640 medium with L-glutamine (2 mM) and penicillin/streptomycin (100 U/mL).

Activin A (100 ng/mL) was added for 3 days, and the defined fetal bovine serum (dFBS) concentration was sequentially adjusted to 0% (day 1), 0.2% (day 2), and 2% (day 3). The differentiation medium was prepared for each use based on the required amount.

####### 2.2.2.1.1.3 Directed differentiation into HG

After DE stages, PSCs were cultured in DMEM/F12 medium containing dFBS (2%), L-glutamine (2 mM), and penicillin/streptomycin (100 U/mL) for 4–6 days. The medium was supplemented with 500 ng/mL fibroblast growth factor 4 (FGF4) and WNT3A (500 ng/mL) or CHIR99021 (3 μM). The differentiation medium was prepared for each use according to the required amount.

####### 2.2.2.1.1.4 hIO basal medium (intestinal organoid basal medium)

hIO basal medium (intestinal organoid basal medium). hIO basal medium comprised advanced DMEM/F12 medium supplemented with L-glutamine (2 mM), HEPES (10 μM), 1× N2 (optional) and B-27 supplements, and penicillin/streptomycin (100 U/mL). It can be stored at 4°C for up to 1 month.

####### 2.2.2.1.1.5 hIO complete medium (intestinal organoid expansion medium)

hIO complete medium (intestinal organoid expansion medium). To prepare the hIO complete medium, the basal medium was supplemented with Rspo1 (200–500 ng/mL), noggin (40–100 ng/mL), and EGF (100 ng/mL).

Note: The concentration ranges of Rspo1 and noggin may vary based on the diversity of the target cells and research objectives.

###### 2.2.2.1.2 Growth factors

The recombinant human growth factors used in the generation of DE, HG, hIOs, and Mat-hIOs were Activin A; FGF4 and WNT3A or CHIR99021; Rspo1, Noggin, and EGF; and IL-2, Rspo1, noggin, and EGF, respectively.

###### 2.2.2.1.3 Reagents

For the generation of hPSCs, DE, HG, and hIOs the reagents used were mTeSR™1 complete kit, Y-27632 Rho kinase inhibitor, dispase or accutase, Matrigel or other ECM; RPMI 1640 medium supplemented with dFBS, L-glutamine, and penicillin/streptomycin; DMEM/F12, dFBS, L-glutamine, and penicillin/streptomycin; and Matrigel or other ECM (Kim et al., 2022; Li et al., 2024), advanced DMEM/F12, B-27 and N2 supplements, L-glutamine, HEPES, and penicillin/streptomycin, respectively.

##### 2.2.2.2 Culture process and requirements

###### 2.2.2.2.1 Culture protocols

####### 2.2.2.2.1.1 Maintenance of hPSCs

Our research complies with all relevant ethical regulations. All studies based on human pluripotent stem cells were approved by the Korean Public IRB (IRB numbers: P01-201409-ES-01-09, P01-201609-31-002).

1. The hESCs and hiPSCs were maintained in mTesR1 medium in Matrigel-coated culture dishes.
2. Cells were passaged approximately every 4 days, based on their density.
3. For passaging, cells were washed with DMEM/F12 (serum-free) and cultured in DMEM/F12 containing dispase or accutase (1 mg/mL) until the colony edges began to separate from the dish.
4. The cells were washed liquid nitrogen twice with DMEM/F12, and the medium was replaced with mTeSR1 after the final wash.
5. Colonies were scraped or gently pipetted into small clumps.
6. The cells were then passaged onto fresh Matrigel-coated dishes.
7. On day 0, cells were cultured in mTeSR1 medium supplemented with 10 μM Y27632.
8. On day 1, the medium was replaced with mTeSR1 and the cells were cultured in a 5% CO_2_ cell culture incubator at 37°C.

####### 2.2.2.2.1.2 Differentiation of hPSCs into DE

1. For organoid differentiation, cells were passaged at a density higher than the maintenance density of hPSCs. Organoid differentiation was initiated after reaching 80% confluence on day 2 of cell growth.
2. The Activin A differentiation protocol was followed for 3 days. hPSCs were treated with Activin A (100 ng/mL) in RPMI 1640 medium for three consecutive days, increasing the concentration of dFBS from 0% to 2% every 24 h.

####### 2.2.2.2.1.3 Directed differentiation into HG

1. The medium was replaced with HG medium on day 4 of differentiation from DE.
2. The cells were cultured in DMEM/F12 medium supplemented with dFBS (2%), FGF4 (500 ng/mL), and WNT3A (500 ng/mL) or CHIR99021 (3 μM) for 4–6 days.
3. The medium was changed every 2 days.

####### 2.2.2.2.1.4 Directed differentiation into hIOs

1. After 4–6 days of HG growth factor treatment, 3D floating spheroids were generated in the culture.
2. Spheroids were inserted into Matrigel.
3. Following the solidification of Matrigel, spheroids were transferred into hIO basal medium containing L-glutamine, HEPES (10 μ), N2 (optional) and B-27 supplements, and penicillin/streptomycin. The hIO complete medium was supplemented with growth factors (200–500 ng/mL Rspo1, 40–100 ng/mL noggin, and 100 ng/mL EGF).
4. The medium was changed every 2–4 days.

####### 2.2.2.2.1.5 Passaging

1. After 10–14 days of intestinal organoid culture, the diameter of the organoids ranged between 0.5 and 2 mm.
2. The intestinal organoids were gently separated from the Matrigel dome by pipetting up and down. This step resulted in the separation of organoids, and any residual Matrigel pieces around the organoids were removed.
3. The intestinal organoids were mechanically divided using a surgical blade or chopper at a ratio of 1:3 to 1:6.
4. The divided pieces of intestinal organoids, including the crypt structures were collected.
5. The lumen of intestinal organoids was washed with advanced DMEM/F12, and dead cells were removed.
6. Undiluted Matrigel (45 μL) was added to a 4-well dish to form a Matrigel dome.
7. Five to seven small pieces of intestinal organoids were inserted into the Matrigel dome and incubated at 37°C with 5% CO_2_ for 10 min to allow Matrigel dome solidification.
8. An additional 5 μL of Matrigel was added to cover the dome.
9. Intestinal organoid expansion medium (700 μL) was added at 37°C and the medium was replaced every 2 days.

####### 2.2.2.2.1.6 Maturation of hIOs induced by interleukin 2

1. When 3D floating spheroids (HG) were formed in the culture, they were inserted into Matrigel. This passage was referred to as passage 0 (P0) for the hIOs.
2. Following Matrigel solidification, the spheroids were placed in an intestinal organoid basal medium containing L-glutamine, HEPES (10 μM), and B-27 supplements, and penicillin/streptomycin. For intestinal organoid expansion, the medium was supplemented with growth factors (200–500 ng/mL Rspo1, 40–100 ng/mL noggin, and 100 ng/mL EGF).

Note: The concentration ranges of Rspo1 and noggin may vary based on the diversity of the target cells and research objectives.

3. The medium was changed every 2–4 days. The cultures were treated daily with fresh IL-2 (1 ng/mL) from passage 0 (P0) to passage 2 (P2).
4. After 10–14 days of intestinal organoid culture, passaging was performed using the traditional intestinal organoid passaging method.
5. The intestinal organoid expansion medium was changed every 2 days.

###### 2.2.2.2.2 Essential environmental conditions

The culture was maintained at 37°C in a 5% CO_2_ incubator with saturating humidity. All culture steps were performed in a sterile environment to prevent contamination. Additionally, after the HG stage, it was essential to culture hPSC-derived organoids for a minimum of 28 days to obtain ISC characteristics.

Note: At 28 days, the organoids exhibited the absence of G-protein-coupled receptor 5 (LGR5) expression and broad expression of Achaete-scute complex homolog 2 (ASCL2). They were not restricted to SOX9+ proliferative zones. However, organoids cultured for up to 56 days exhibited the co-expression of ASCL2 and LGR5 in a limited epithelial domain overlapping with SOX9+ zones.

### 2.3 Quality requirements and assessment

#### 2.3.1 Organoid critical quality attributes (CQAs)

##### 2.3.1.1 Morphological quality

###### 2.3.1.1.1 Adult stem cell-derived intestinal organoids (hASC-IOs)

The hASC-IOs exhibited a bud-like morphology with a central lumen and retained a circumferential epithelial cell structure (Sato et al., 2009; Sato et al., 2011a; Wang R. et al., 2022).

###### 2.3.1.1.2 Pluripotent stem cell-derived intestinal organoids (hPSC-IOs)

The size of the hPSC-IOs ranged from 0.5 to 2 mm. They exhibited bud-like structures and featured a central lumen that closely interacted with circumferential epithelial cells. The formation of highly intricate epithelial structures was facilitated by the surrounding primitive mesenchyme (intestinal stromal cells) (Spence et al., 2011).

###### 2.3.1.1.3 Matured hPSC-IOs (Mat-hIOs)

Matured hPSC-IOs were larger (1–2 mm) than that of the hPSC-Ios, exhibiting numerous and complex bud-like structures. They formed an intricate epithelial structure surrounded by the primitive mesenchyme (intestinal stromal cells) (Jung et al., 2018).

##### 2.3.1.2 Various cell types

###### 2.3.1.2.1 Adult stem cell-derived intestinal organoids (hASC-IOs)

The hASC-IOs consisted of ISCs, TA cells, enterocytes, goblet cells, and enteroendocrine cells (Tetteh et al., 2016).

###### 2.3.1.2.2 Pluripotent stem cell-derived intestinal organoids (hPSC-IOs)

The hPSC-IOs consisted of ISCs, TA cells, enterocytes, goblet cells, Paneth cells, and enteroendocrine cells. They were enveloped by the primitive mesenchyme (intestinal stromal cells) (Spence et al., 2011).

###### 2.3.1.2.3 Matured hPSC-IOs (Mat-hIOs)

Olfactomedin-4 (OFLM4+) is expressed in ISCs, Defensin Alpha 5 (DEFA5+) in Paneth cells, and mucin 13 (MUC13+) in goblet cells. Additionally, Keratin 20 (KRT20) was expressed in the mature epithelium of the crypt (Jung et al., 2018).

Note: The proportion of LGR5+ or ASCL2+ ISCs; KI67+ and LGR5− TA cells; alkaline phosphatase inhibitor (ALPI+), Villin+, or intestinal fatty acid binding protein (IFABP+) enterocytes; MUC2+ goblet cells; lysozyme (LYZ+) or matrix metallopeptidase 7 (MMP7+) Paneth cells; and chromogranin A (CHGA+) enteroendocrine cells in the intestinal epithelial cells should be a minimum of 30%.

##### 2.3.1.3 Organ-specific functionality

###### 2.3.1.3.1 Intestine-specific markers

• LGR5+ or ASCL2+: ISCs (Barker et al., 2007)
• LGR5-: TA cells (Beumer and Clevers, 2021)
• ALPI+, Villin+ or IFABP+: Enterocytes (Tetteh et al., 2016)
• MUC2+: Goblet cells (Chang et al., 1994)
• CHGA+: Enteroendocrine cells (Zeve et al., 2022)
• Ki67+: Proliferating cells (Beumer and Clevers, 2021)
• LYZ+ or MMP7+: Paneth cells (Bevins and Salzman, 2011)

###### 2.3.1.3.2 Mature intestine-specific markers

• LGR5+, ASCL2+ or OLFM4+: ISCs
• LGR5-: TA cells
• KRT20+: Enterocytes in the villi
• ALPI+, Villin+ or IFABP+: Enterocytes
• MUC2+ or MUC13+: Goblet cells
• CHGA+: Enteroendocrine cells
• Ki67+: Proliferating cells
• LYZ+, MMP7+ or DEFA5+: Paneth cells
• Among the functional markers, dipeptidyl-peptidase 4 (DPP4) and lactase (LCT) are associated with intestinal digestive function, SLC5A1 also known as sodium-dependent glucose transporter (SGLT1), serves as a glucose transporter, sucrase-isomaltase (SI) serves as a functional brush-border enzyme, and multidrug resistance 1 (MDR1) and peptide transporter 1 (PEPT1) serve as intestinal transporters (Jung et al., 2018).

###### 2.3.1.3.3 Intestinal alkaline phosphatase (IAP): ALPI test (enterocyte function)

IAP is an intrinsically expressed protein in the intestinal epithelium that plays a crucial role in maintaining intestinal homeostasis (Sensoy and Oznurlu, 2019; Wang et al., 2020).

###### 2.3.1.3.4 Mucin: mucin staining (goblet cell function)

Mucin is a highly glycosylated protein that protects epithelial cells. Muc2, secreted by goblet cells in the small intestine and colon, is a significant component of the loose mucus layer that entraps luminal material (Chang et al., 1994).

##### 2.3.1.4 Chromosomal karyotype and identification

###### 2.3.1.4.1 Chromosomal karyotype: 46, XY or 46, XX

Karyotype analysis was used to assess the size, shape, and number of chromosomes in the cell samples from the organoids, ensuring the presence of a complete set of 46 chromosomes (Sato et al., 2011a).

###### 2.3.1.4.2 Identity: STR profiling, CNVs

Short Tandem Repeat (STR) loci are highly informative human genome markers. STR profiling ensures the quality and integrity of intestinal organoids (Lee et al., 2015).

Copy Number Variations (CNVs) identify genetic variations in intestinal organoids and reflect structural changes that result in abnormal copy numbers of one or more genes.

#### 2.3.2 Organoid quality assessment endpoints

##### 2.3.2.1 Size and morphological features

hIOs exhibited a bud-like morphology with a central lumen and circumferential epithelial cell structure. The total cell count in the hPSC-IOs ranged from 1 × 10^4^–2 × 10^5^ cells/organoid. Their size ranged from 0.5–2 mm.

##### 2.3.2.2 Essential cell types

• ISCs (LGR5+ or ASCL2+), TA cells (LGR5-), goblet cells (MUC2+), enterocytes (ALPI+ or Villin+ or IFABP+), and proliferating cells (Ki67) were commonly observed
• Specifically, hASC-IOs comprised of ISCs, TA cells, enterocytes, goblet cells, and enteroendocrine cells, with a minimum of 30% enterocytes
• The hPSC-IOs included ISCs, TA cells, enterocytes, goblet cells, Paneth cells, and enteroendocrine cells, with a minimum of 30% enterocytes

##### 2.3.2.3 Specific gene expression and functional testing

###### 2.3.2.3.1 Specific gene expression (gene and protein expression)

• Cell type marker gene analysis (Real-time Fluorescence Quantitative PCR Method) compared target protein with glyceraldehyde 3-phosphate dehydrogenase (GAPDH)
• Cell composition ratio analysis (Immunofluorescence Staining Method) compared the target protein with 4′,6-diamidino-2-phenylindole (DAPI)

Note: Cell type markers: LGR5 and ASCL2 for ISCs; MUC2 for goblet cells; ALPI, Villin, and IFABP for enterocytes; CHGA for enteroendocrine cells; MKI67 for proliferating cells. Paneth cell marker (LYZ) was detected in colonic organoids. Additionally, hPSC-derived intestinal organoids expressed CDX2, KLF5, and SOX9.

###### 2.3.2.3.2 Functional assays

• Goblet cell function assessment:Mucin test (Mucin staining method)
• AP, Lysozyme measurement:Alkaline phosphatase and lysozyme staining
• Organoid viability assessment:Calcein-acetoxymethylester (Calcein-AM) staining method
• Permeability testing: Fluorescein isothiocyanate (FITC)-dextran 4 kDa, 40 kDa assays
• P-glycoprotein (P-gp)/MDR1 activity: P-gp/MDR1 activity assay
• GIP measurement: Hormone secretion assay, ELISA
• Absorption function assessment: Forskolin assay

#### 2.3.3 Methods for each quality control (QC) metrics

##### 2.3.3.1 Morphology

Under differential interference microscopy, the intestinal organoids were observed. Their size ranged from approximately 0.2–2 mm, displaying a bud-like structure with a central lumen and an epithelial cell layer.

##### 2.3.3.2 Cell numbers

Cell observations were conducted using a hemocytometer or cell counter. After dissociating organoids from Matrigel, they were rinsed with 4°C phosphate-buffered saline (PBS) or advanced DMEM/F12 medium. Cells were disaggregated using ethylenediaminetetraacetic acid (EDTA), washed liquid nitrogen twice with advanced DMEM/F12, and measured using a hemocytometer or cell counter.

##### 2.3.3.3 Various cell types (immunocytochemistry and immunofluorescence staining)

The intestinal organoids were prepared using 4% paraformaldehyde (PFA). Whole organoids or sections (frozen or paraffin-embedded) were permeabilized using 0.1% Triton X-100 for 15 min. Subsequently, the cells were treated with 4% BSA, followed by sequential incubation with primary (target protein) and secondary antibodies (incubation times and storage temperatures may vary based on the antibody type). The nuclei were stained with DAPI, and the samples were examined under a microscope. The ratio of cells positive for the target protein (N) to the total cell count (M) was calculated and is represented as X = N/M.

##### 2.3.3.4 Marker genes (real-time fluorescence quantitative PCR)

Total RNA was extracted using commercial RNeasy kits or the TRIzol assay, followed by reverse transcription using a commercial cDNA synthesis kit. qPCR was performed using a Real-time PCR system (for three independent samples within the experimental group). RNA extracted from the commercial human intestine (small or large intestine) was used as a positive control and GAPDH served as a housekeeping gene control. The average expression of the target genes was calculated using three replicates. Expression of the target gene (*CtM*) relative to that of GAPDH (*CtG*) was calculated as the 2^−ΔΔCT^ method.

##### 2.3.3.5 Organ-specific function

###### 2.3.3.5.1 ALPI detection: commercial alkaline phosphatase chromogenic kit or immunocytochemistry

The medium was removed from the cultured organoids, rinsed liquid nitrogen twice with an equal volume of PBS, and fixed with 4% PFA. ALPI staining was performed following to the manufacturer instructions. Detection was assessed based on staining intensity.

###### 2.3.3.5.2 Mucin detection: commercial AB-PAS or mucin staining kit

The medium was removed from the cultured organoids, rinsed liquid nitrogen twice with an equal volume of PBS, and fixed with 4% PFA. AB-PAS or mucin staining was performed following the manufacturer’s instructions. Detection was assessed based on the staining intensity.

###### 2.3.3.5.3 Absorptive function: swelling assay

A culture medium containing forskolin (20 mM) and DMSO was prepared, each added at 0.1% (v/v). The culture medium containing forskolin or DMSO was added to the organoids and treated for approximately 10–12 h at 37°C in a CO_2_ incubator. Organoid swelling was observed under a microscope by comparing the test group (forskolin-treated) with the negative control group (DMSO-treated) (Boj et al., 2017; Vonk et al., 2020).

##### 2.3.3.6 Chromosomal karyotype and STR analysis

###### 2.3.3.6.1 Sample preparation for chromosomal karyotype and STR

Organoids were isolated from the Matrigel using a pipette, washed with PBS, and collected through centrifugation.

###### 2.3.3.6.2 Chromosomal karyotype

Chromosomes were isolated from cell nuclei, stained using specific methods, and mounted on slides. Subsequently, the chromosomes were photographed under a microscope and arranged in pairs using the jigsaw puzzle method. They were organized by size from 1 to 22, followed by the sex chromosomes as the 23rd pair. Abnormalities were analyzed using chromosome pair analysis.

###### 2.3.3.6.3 Copy number variation (CNV)

CNV testing revealed fine-scale DNA copy number variations that were not detected through chromosomal karyotype analysis. It was commonly analyzed using chromosomal microarrays or next-generation sequencing (NGS).

###### 2.3.3.6.4 STR analysis

STR analysis was performed at ten different loci on different chromosomes to confirm the presence of organoid samples. The target DNA was amplified through PCR using fluorescent-labeled primers, and the resulting DNA was assessed using a DNA sequencer.

##### 2.3.3.7 Viability (3D cell viability assay)

###### 2.3.3.7.1 hASC-IOs (small-sized organoids)

Viability testing was performed using calcein-AM. Organoid size, shape, and conditions were observed under a microscope. Organoids that met the final requirements (diameter ≥200 μm) were counted. The solution was incubated with calcein-AM stock solution at a final concentration of 0.2 μmol/L for 60 min at 37°C. Live organoids (diameter ≥ 200 μm) exhibiting fluorescence (green) were counted under a fluorescence microscope (excitation: 490 nm, emission: 515 nm).

###### 2.3.3.7.2 hPSC-IOs (large-sized organoids)

Cell viability was assessed using a 3D cell viability assay. Organoids were cultured in an opaque multi-well plate and treated with compounds following to the manufacturer’s instructions. The experimental conditions were optimized based on sample volume and microtissue characteristics. Following staining, live organoids emitting fluorescence were assessed using a fluorescence microscope.

#### 2.3.4 Monitoring of quality assessment results (batch and periodic analysis)

1. Cultivation and Growth: Organoids should be sub-cultured to at least 1:3 or more.
2. Microorganisms: They should test negative for algae, bacteria, *mycoplasma*, and viruses.
3. Identity: Should match the first generation through STR analysis.
4. In general, the same quality assessment tests were performed liquid nitrogen twice.

### 2.4 Storage and preservation

#### 2.4.1 Storage protocol

The cultured cells were incubated at 37°C with 5% CO_2_ under saturating humidity. For long-term preservation, they were stored in liquid nitrogen (−196°C) or at ultra-low temperatures (Pleguezuelos-Manzano et al., 2020; Lee et al., 2022).

Note: The storage duration should be based on stability tests (refer to Section 2.3.4).

##### 2.4.1.1 Freezing and thawing process

###### 2.4.1.1.1 Freezing

1. Intestinal organoids were separated from the Matrigel or BME domes and collected in a 60 mm cell culture dish.
2. The samples were mechanically chopped using a tissue chopper.
3. The chopped organoids were collected, and 1 mL of 4°C PBS was added and pipetted gently to remove solid Matrigel pieces.
4. They were washed liquid nitrogen twice with 1 mL of 4°C DMEM/F12 containing 15 mM HEPES and pelleted using a benchtop centrifuge.
5. The supernatant was carefully removed, and the pellet was resuspended in 1 mL of cold (2°C–8°C) freezing medium per 30–50 organoid pieces.
6. The organoid pellet was transferred to labeled cryovials using a P1000 pipette.
7. The cryovials containing organoids were placed in a freezing container with isopropanol and stored at −80°C for 24 h.
8. The cryovials were transferred to liquid nitrogen (−196°C) for long-term storage.

For hASC-IOs, 10 mL of DMSO (Sigma-Aldrich, Catalog Number D2650) and 40 mL of FBS (Sigma-Aldrich, Catalog Number F7524) were used. For organoids derived from hPSCs, a commercially available freezing medium without FBS was used.

###### 2.4.1.1.2 Thawing

1. A warm (37°C) basic medium was prepared for intestinal organoids and proliferation medium.
2. The frozen organoids were thawed by placing the cryovial in a 37°C water bath.
3. Once the freezing medium turned semi-liquid, it was removed from the water bath, and basic medium (1 mL) was added to complete thawing.
4. The contents of the cryovial were transferred to a 15 mL tube and up to 10 mL of basic medium was added.
5. Subsequently, the contents were centrifuged (1,250 rpm, 5 min). The supernatant was carefully removed, and the pellet was resuspended in the proliferation medium (100 μL).
6. Matrigel (100 μL) was added using a P200 pipette, and the suspension was gently mixed by pipetting up and down.
7. Domes were generated using an organoid suspension (45 μL) in a 4-well multi-dish. The cells were incubated at 37°C in 5% CO_2_ for 10 min for Matrigel solidification.
8. Five microliters of Matrigel or BME was added to cover the domes after solidification.
9. A warm proliferation medium (700 μL) supplemented with 10 μM Y-27632 was added to the dish.
10. After 24 h, the medium was replaced with the proliferation medium without Y27632.

##### 2.4.1.2 Essential equipment and instruments

Tissue choppers, surgical blades, cryogenic tubes, freezing containers, cryoboxes, and liquid nitrogen tanks.

#### 2.4.2 Post-storage QC factors

After a defined storage period, it was crucial to assess whether the organoids retained their intrinsic properties and characteristics following freezing and thawing. Establishing the factors and criteria for assessing the quality of post-storage organoids is essential. Although the factors for assessing organoid quality following storage may vary based on the organoid type and intended use, common elements are recommended for organoid quality assessment. They include viability, microorganism testing, morphology, genetic stability, gene expression, proliferation and growth, and functional assays.

##### 2.4.2.1 Post-thaw viability

The post-thaw viability of the organoids should be a minimum of 50%, and these viable organoids should be further sub-cultured in test tubes.

##### 2.4.2.2 Microorganism testing

Organoids should test negative for fungi, bacteria, mycoplasmas, and viruses.

##### 2.4.2.3 Cell identification

**FIGURE 2:**
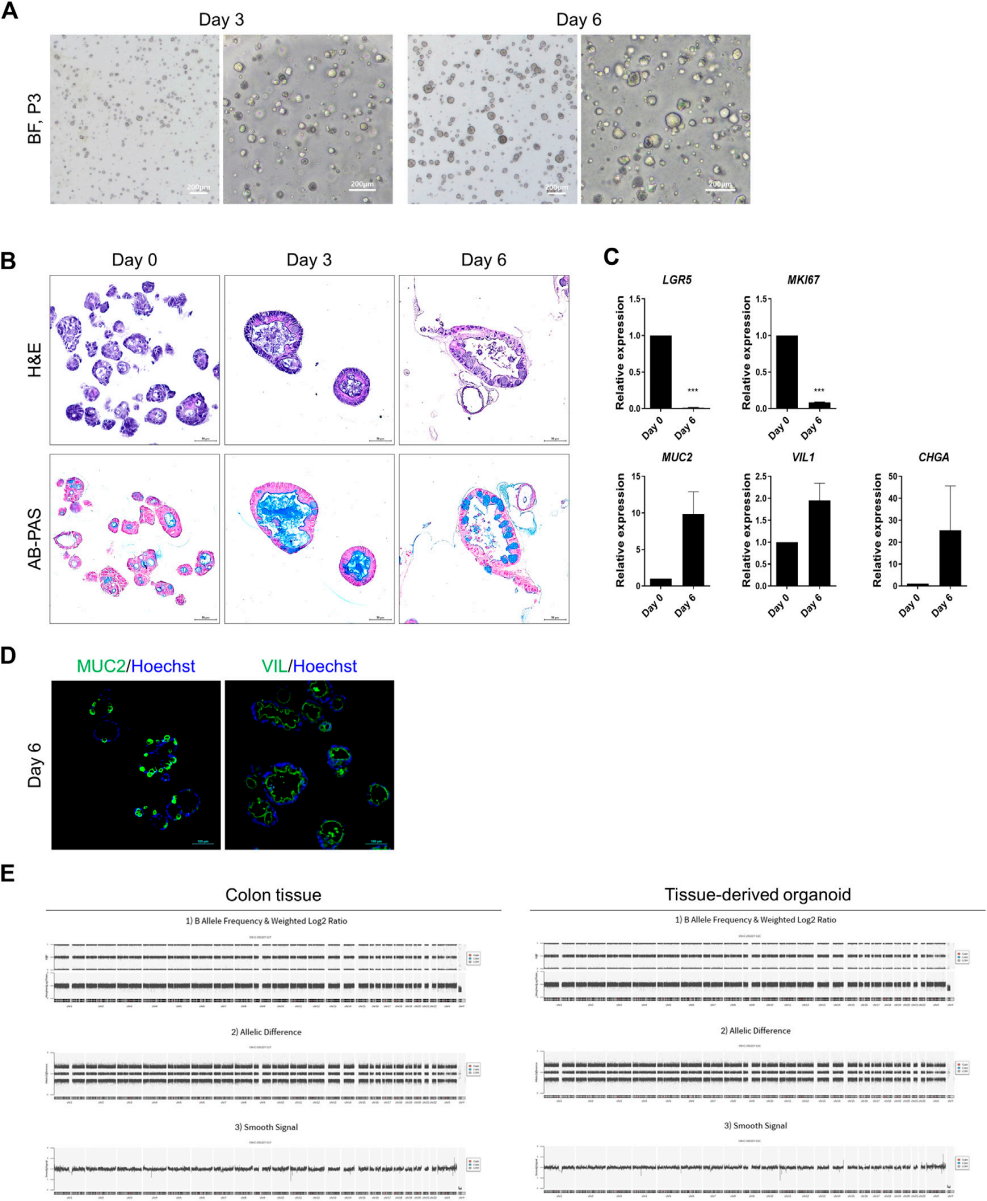
Representative data illustrating the cell characteristics and identification of hASC-derived organoids. **(A)** Bright-field microscopy images of hASC-derived organoids on day 3 (left) and on day 6 of passage 3 (right). Scale bar in bright-field microscopy images, 200 μm. **(B)** Histological analysis using hematoxylin and eosin staining (top) and alcian blue staining (bottom). Scale bar, 50 μm. **(C)** Specific-gene expression of *LGR5* (ISCs), Ki67 (*MKI67*, proliferating cells), mucin 2 (*MUC2*, goblet cells), villin1 (*VIL1*, enterocytes) and chromogranin A (*CHGA*, enteroendocrine cells) in hASC-derived organoids on day 0 and day 6 by qRT-PCR assay. Each gene expression was normalized by GAPDH expression. Bars show mean and SEM (*n* = 3). **(D)** Immunofluorescence staining of MUC2 (left, green) and VIL (right, green) from colon organoids differentiated for 6 days. Nuclei were stained with hoechst (blue). Scale bar, 100 μm. **(E)** Whole genome copy number variation analysis of colon tissue and organoids from human adult stem cells. Colon tissue (left), tissue derived organoid (right). Segments are filtered by relevant cancer markers, contain pathogenic regions not reported in the Database of Genomic Variants, and losses or gains satisfying at least 25 kb in size and MarkerCount greater than 10. There are no abnormal segments in sample.

**FIGURE 3:**
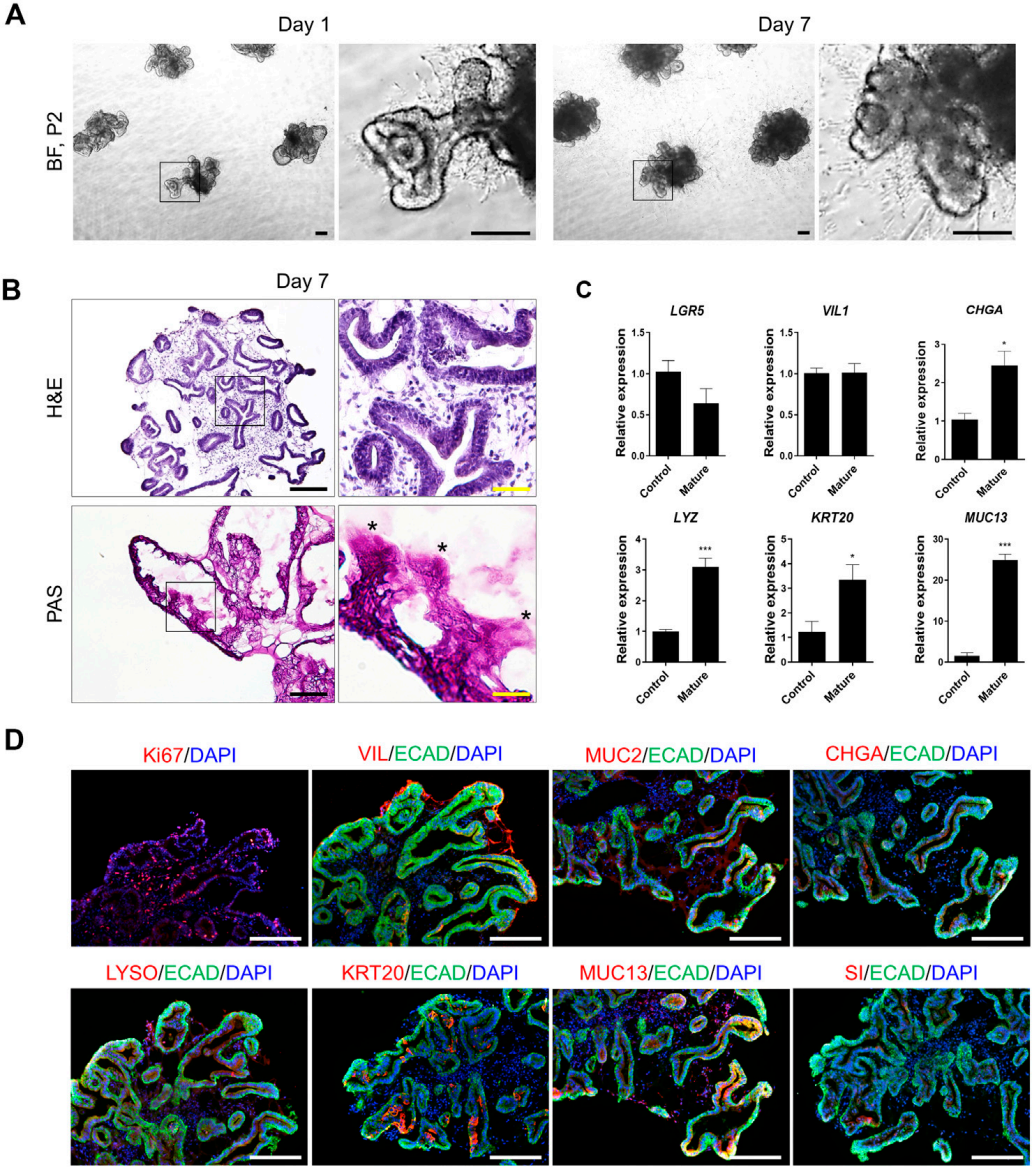
Representative data illustrating the cell characteristics and identification of hPSC-derived mature intestinal organoids. **(A)** Bright-field microscopy images of hPSC-derived organoids on day 1 (left) and day 7 (right) of passage 2. Scale bar, 200 μm. **(B)** Histological analysis using hematoxylin and eosin staining (top) and PAS staining (bottom). Scale bar, black 200 μm and yellow 50 μm. **(C)** Specific-gene expression of *LGR5* (ISCs), *VIL1* (enterocytes), *CHGA* (enteroendocrine cells)*, LY*Z (paneth cells)*, KRT20* and *MUC13* (mature intestinal markers) in control and mature hIOs by qRT-PCR assay. Each gene expression was normalized by GAPDH expression. Bars show mean and SEM (*n* = 4). **(D)** Immunofluorescence staining of Ki67, VIL, MUC2, CHGA, LYSO, KRT20, MUC13 and Sucrase isomaltase (SI) in hPSC-derived mature intestinal organoids at 7 days. Nuclei were stained with DAPI (blue). Scale bar, 275 μm.

Appropriate tests were conducted to identify the cells produced by the cell bank. Morphological and functional analyses are beneficial when combined with other tests. Cell sources from various donors were differentiated by assessing genomic polymorphism patterns using CNV or STR analyses. Morphological analyses were performed using differential interference contrast microscopy. Specific staining of the intestine, such as ALPI and mucin staining, is used in functional tests in addition to immunostaining and qPCR (see Figures 2, 3).

## Figures and Tables

**Table:** 

**hASCs**	human adult stem cells
**hPSCs**	human pluripotent stem cells
**hESCs**	human embryonic stem cells
**hiPSCs**	human-induced pluripotent stem cells
**iPSCs**	induced pluripotent stem cells
**HIV**	Human Immunodeficiency Virus
**HBV**	Hepatitis B Virus
**HCV**	Hepatitis C Virus
**HEPES**	N-2-hydroxyethylpiperazine-N′-2-ethanesulfonic acid
**MAPK**	mitogen-activated protein kinases
**FBS**	fetal bovine serum
**ALK5**	activin receptor-like kinase 5
**PGE2**	prostaglandin E2
**Rspo1**	R-Spondin1
**BME**	basement membrane extracts
**ECM**	extracellular matrix
**RPMI**	Roswell Park Memorial Institute
**dFBS**	defined fetal bovine serum
**FGF4**	fibroblast growth factor 4
**DE**	definitive endoderm
**PSCs**	pluripotent stem cells
**IL-2**	interleukin-2
**LGR5**	G-protein-coupled receptor 5
**ASCL2**	Achaete-scute complex homolog 2
**hASC-IOs**	adult stem cell-derived intestinal organoids
**hPSC-IOs**	pluripotent stem cell-derived intestinal organoids
**Mat-hIOs**	matured huma intestinal organoids
**OFLM4+**	Olfactomedin-4
**DEFA5+**	Defensin Alpha 5
**MUC13+**	Mucin 13
**KRT20**	Keratin 20
**ALPI+**	alkaline phosphatase inhibitor
**IFABP+**	intestinal fatty acid binding protein
**MMP7+**	matrix metallopeptidase 7
**CHGA+**	chromogranin A
**LYZ+**	Lysozyme
**DPP4**	dipeptidyl-peptidase 4
**LCT**	Lactase
**SGLT1**	sodium-dependent glucose transporter
**PEPT1**	peptide transporter 1
**SI**	sucrase isomaltase
**MDR1**	multidrug resistance 1
**IAP**	intestinal alkaline phosphatase
**calcein-AM**	Calcein-acetoxymethylester
**STR**	short tandem repeat
**CNVs**	copy number variations
**GAPDH**	glyceraldehyde 3-phosphate dehydrogenase
**DAPI**	4′,6-diamidino-2-phenylindole
**FITC**	fluorescein isothiocyanate
**PBS**	phosphate-buffered saline
**EDTA**	ethylenediaminetetraacetic acid
**PFA**	paraformaldehyde
**NGS**	next-generation sequencing
**CQAs**	critical quality attributes
**QC**	quality control

## Data Availability

The CNV data presented in the study are deposited in the dbVar repository, accession number is snstd242, available at the following link: https://ftp.ncbi.nlm.nih.gov/pub/dbVar/data/Homo_sapiens/by_study/genotype/nstd242/.
