# Supplementary material for: Standardization and quality assessment for human intestinal organoids
Source: Front Cell Dev Biol. 2024 Sep 12;12:1383893. doi: 10.3389/fcell.2024.1383893 (PMC11424408; doi:10.3389/fcell.2024.1383893)
Supplement: Supplementary file 1 [file Table1.pdf]

Supplementary table 1 Example of qPCR markers for hASC-IOs

hASC-IOs		
**Target**	**TaqMan ID**	**Marker Type**
GAPDH	Hs99999905_m1	All
LGR5	Hs00969422_m1	ISCs
MKI67	Hs01032443_m1	Proliferating cells
MUC2	Hs03005103_g1	Goblet cells
VIL1	Hs00200229_m1	Absorptive cells (Enterocytes)
CHGA	Hs00900370_m1	Enteroendocrine cells
* TaqMan Gene Expression Master Mix, Thermo#4369016		
