# Supplementary material for: Standardization and quality assessment for human intestinal organoids
Source: Front Cell Dev Biol. 2024 Sep 12;12:1383893. doi: 10.3389/fcell.2024.1383893 (PMC11424408; doi:10.3389/fcell.2024.1383893)
Supplement: Supplementary file 2 [file Table2.pdf]

Supplementary table 2 Example of qPCR primer sequences for hPSC-IOs

hPSC-IOs			
**Target**	**Primer sequence**		**Marker Type**
GAPDH	F	GAAGGTGAAGGTCGGAGTC	All
	R	GAAGATGGTGATGGGATTTC	
LGR5	F	TGCTCTTCACCAACTGCATC	ISCs
	R	CTCAGGCTCACCAGATCCTC	
VIL1	F	AGCCAGATCACTGCTGAGGT	Enterocytes
	R	TGGACAGGTGTTCCTCCTTC	
CHGA	F	TGACCTCAACGATGCATTTC	Enteroendocrine cells
	R	CTGTCCTGGCTCTTCTGCTC	
LYZ	F	AAAACCCCAGGAGCAGTTAAT	Paneth cells
	R	CAACCCTCTTTGCACAAGCT	
KRT20	F	TGGCCTACACAAGCATCTGG	Enterocytes (Differentiated cells)
	R	TAACTGGCTGCTGTAACGGG	
